# Study of *Triticum aestivum* Resistome in Response to *Wheat dwarf India Virus* Infection

**DOI:** 10.3390/life11090955

**Published:** 2021-09-13

**Authors:** Jitendra Kumar, Krishan Mohan Rai, Shahryar F. Kianian, Sudhir P. Singh

**Affiliations:** 1 National Agri-Food Biotechnology Institute, SAS Nagar, Mohali 140306, India; jkumar@umn.edu; 2 Department of Agronomy and Plant Genetics, University of Minnesota, St. Paul, MN 55108, USA; 3 Department of Microbial and Plant Biology, University of Minnesota, St. Paul, MN 55108, USA; Kmohanra@umn.edu; 4 USDA-ARS Cereal Disease Laboratory, St. Paul, MN 55108, USA; 5 Center of Innovative and Applied Bioprocessing, SAS Nagar, Mohali 140306, India

**Keywords:** wheat, susceptible, resistant, transcriptome, biotic stress, viruses

## Abstract

Susceptible and resistant germplasm respond differently to pathogenic attack, including virus infections. We compared the transcriptome changes between a resistant wheat cultivar, Sonalika, and a susceptible cultivar, WL711, to understand this process in wheat against wheat dwarf India virus (WDIV) infection. A total of 2760 and 1853 genes were differentially expressed in virus-infected and mock-inoculated Sonalika, respectively, compared to WL711. The overrepresentation of genes involved in signaling, hormone metabolism, enzymes, secondary metabolites, proteolysis, and transcription factors was documented, including the overexpression of multiple PR proteins. We hypothesize that the virus resistance in Sonalika is likely due to strong intracellular surveillance via the action of multiple PR proteins (PR1, RAR1, and RPM1) and ChiB. Other genes such as PIP1, LIP1, DnaJ, defensins, oxalate oxidase, ankyrin repeat protein, serine-threonine kinase, SR proteins, beta-1,3-glucanases, and O-methyltransferases had a significant differential expression and play roles in stress tolerance, may also be contributing towards the virus resistance in Sonalika. In addition, we identified putative genes with unknown functions, which are only expressed in response to WDIV infection in Sonalika. The role of these genes could be further validated and utilized in engineering resistance in wheat and other crops.

## 1. Introduction

Geminiviruses are a large and important family of plant viruses that infect a wide variety of crops worldwide and have been divided into nine genera (*Becurtovirus*, *Begomovirus*, *Capulavirus*, *Curtovirus*, *Eragrovirus*, *Grablovirus*, *Mastrevirus*, *Topocuvirus*, and *Turncurtovirus*) [1]. Studies have been performed to characterize the transcriptional responses of host plants to a geminivirus [2,3,4,5,6], but only a few have explored the transcriptome response of a resistant host plant [7,8].

Wheat dwarf India virus (WDIV) is a monocot infecting mastrevirus with a monopartite genome encoding for two proteins, coat protein (CP) and movement protein (MP), on the virion strand and two replication-associated proteins (Rep and RepA) on the complementary strand [9,10,11]. WDIV has also been found to be associated with satellites and infects barley and sugarcane, in addition to wheat [10,11].

Viral infection triggers a complex interaction between the virus and the host leading to the reprogramming of various physiological and metabolic processes, including gene expression [12]. Understanding host responses in terms of reprogramming of the gene expression during viral infection can help in developing strategies for virus control [7,13,14]. Microarray has been widely and successfully used to study differential gene expression in plants under different abiotic and biotic stresses [15,16], including studies that explore the response of host plants to a geminivirus infection [2,5,17].

In previous studies, we examined several wheat cultivars for their sensitivity to WDIV [10,18]. A wheat cultivar, WL711, appeared to be very sensitive to WDIV infection, showing severe symptoms. On the other hand, the cultivar Sonalika showed very mild symptoms under identical conditions. In this work, we carried out a comparative transcriptome analysis of resistant vs. sensitive cultivars upon WDIV infection.

## 2. Materials and Methods

### 2.1. Germplasm

In this study, the WDIV resistant wheat cultivar, Sonalika, and susceptible cultivar, WL711, were selected for the transcriptome comparison. As documented earlier [10,18], Sonalika shows very mild symptoms, and the WL711 exhibit severe symptoms, such as reductions in plant height, upon WDIV infection.

### 2.2. Virus Inoculation and Plant Growth Conditions

An infectious clone of WDIV from a previous study [10] was transformed into the *Agrobacterium tumefaciens* strain GV3101 via the freeze-thaw method. A recombinant *A. tumefaciens* colony containing the desired construct was selected and grown in 5 mL Luria-Bertani (LB) medium for 48 h at 28 °C and 200 rpm in a rotary shaker. About 500 μL primary culture was inoculated in 200 mL fresh LB medium and incubated overnight at 28 °C and 200 rpm. Upon achieving the optical density of 1 at 550 nm, the cells were harvested and resuspended in 200 mL infiltration buffer (10 mM morpholineethanesulfonic acid [MES], 10 mM MgCl_2_, and 200 μM acetosyringone) and incubated in the dark with shaking for 4 h. Young leaves of the wheat seedlings at the 5–6 leaf stage were wounded by pinching with a needle and then inoculated by dipping in the infiltration buffer containing agrobacterium. A total of six plants from each cultivar, Sonalika and WL711, were inoculated with recombinant *A. tumefaciens* transformed with the virus construct, and three plants from each cultivar were inoculated with untransformed *A. tumefaciens* cells and were included as controls. The inoculated plants were maintained in plant growth chambers (PGR14; Conviron, Winnipeg, MB, Canada) at 22 °C until the completion of the experiment.

### 2.3. Sample Collection and RNA Isolation

All the virus-inoculated plants from each cultivar were tested for the presence of the virus. Total DNA was isolated using a DNeasy Plant Mini Kit (Qiagen, Hilden, Germany) and used in the PCR as a template. WDIV-specific primers [9] were used to test the presence and systemic spread of WDIV in the inoculated plants. Leaf samples were collected from three biological replicates showing the presence of the virus, frozen in liquid nitrogen, and stored at −80 °C until RNA extraction. Total RNA was extracted using a Spectrum Plant Total RNA Kit (Sigma-Aldrich, St. Louis, MO, USA). Any residual DNA was removed using on-column DNase I (Sigma-Aldrich) digestion. RNA integrity was determined using gel electrophoresis.

### 2.4. Microarray Hybridization

For cDNA synthesis, about 200 ng total RNA and oligo(dT)-T7 primer were used in the total reaction volume of 20 μL. Biotinylated amplified RNA (aRNA) was synthesized using the double-stranded cDNA template from the previous step. The aRNA was then purified and fragmented following instructions provided in the Affymetrix manual. A total of 15 μg purified and fragmented aRNA was hybridized to a GeneChip Wheat Genome Array (Affymetrix Part# 900560) for 16 h. GeneChip Fluidic Station 450 was used for washing and staining the arrays, following the EukGe-Wsv4 protocol. The processed arrays were scanned using a GeneChip Scanner (Affymetrix, Santa Clara, CA, USA). Qualities of the arrays were assessed following the Affymetrix recommended parameters.

### 2.5. Differential Gene Expression Analysis

As recommended in previous studies [19,20,21], the microarray analysis was performed using two biological replicates. The data has been deposited to NCBI (http://www.ncbi.nlm.nih.gov, accessed on 7 July 2021), with accession number GSE179675. The CEL files produced from the control (Agrobacterium only) and virus (Agrobacterium transformed with virus construct) inoculated Sonalika (Sonalika_C and Sonalika_V) and WL711 (WL711_C and WL711_V) wheat cultivars were analyzed using Transcriptome Analysis Console (TAC) Software (ThermoFisher Scientific, Waltham, MA, USA). Robust Multiarray Average (RMA) normalized data were log2 transformed and used to obtain fold change value in the following combinations: (i) Sonalika_V vs. WL711_V, (ii) WL711_C vs. Sonalika_C, (iii) Sonalika_V vs. Sonalika_C, and (iv) WL711_V vs. WL711_C. Probesets with fold change value of ≤−2 or ≥2 with a significant *p*-value cutoff of <0.05 were classified as the differentially expressed genes (DEGs). Venn diagrams of up- and down-regulated genes were generated using Venny 2.0 (https://bioinfogp.cnb.csic.es/tools/venny/, accessed on 11 February 2021).

### 2.6. Gene Ontology Analysis

Gene Ontology (GO) enrichment analysis of DEGs from Sonalika_V vs. WL711_V and WL711_C vs. Sonalika_C was performed using the online tool AgriGO v2 (http://systemsbiology.cau.edu.cn/agriGOv2/, accessed on 26 February 2021). Singular Enrichment Analysis (SEA) was performed using default parameters such as Fisher as a statistical test method; Yekutieli (FDR under dependency) as a multi-test adjustment method, and the significance level was set at *p* < 0.05.

### 2.7. MapMan Analysis

To visualize the differentially expressed genes in response to pathogen attack, a selected set of genes were subjected to pathway analysis using the MapMan version 3.5.1R2. MapMan bins for the GeneChip^®^ Wheat Genome Array was downloaded from the Affymetrix NetAffix analysis center (https://www.affymetrix.com/analysis/netaffx/showresults.affx, accessed on 13 February 2021). The expression heat map of DEGs mapped to various pathways such as abiotic and biotic stress, TFs, hormones, enzymes, secondary metabolites, and protein synthesis and degradation was created using the MeV v2.0 [22].

### 2.8. KEGG Pathway Analysis

For the KEGG pathway analysis of the DEGs, parent nucleotide sequences of the microarray probesets were assigned to the corresponding *Triticum aestivum* gene id using Blastn [23]. KEGG’s annotation tool BlastKOALA (https://www.kegg.jp/blastkoala/, accessed on 10 July 2021) was used to assign the K numbers to the *T. aesitvum* genes representing differentially expressed probe sets. The assigned K numbers were used to perform the pathway analysis using the KEGG pathway database (https://www.genome.jp/kegg/pathway.html, accessed on 10 July 2021).

### 2.9. Quantitative Real Time PCR

cDNA was synthesized using the RNA samples and SuperScript III First Strand cDNA Synthesis Kit (Life Technologies, Carlsbad, CA, USA). Quantitative real-time PCR was performed using SYBR Green (Qiagen, Hilden, Germany) fluorescence dye and analyzed by a 7500 Fast Real-Time PCR System (Applied Biosystems, Waltham, MA, USA). The real-time PCR-based validations of randomly selected genes were performed in three replicates. Gene-specific primers (Table S1) were used in the PCR reactions. The elongation factor-1 alpha (EF-1αfor/rev; Table S1) was used as an internal control to normalize the expression data, and the 2^−ΔΔCt^ method was used to calculate the relative fold change in the gene expression.

## 3. Results and Discussion

### 3.1. Virus Detection and Symptom Analysis

**Figure 1:**
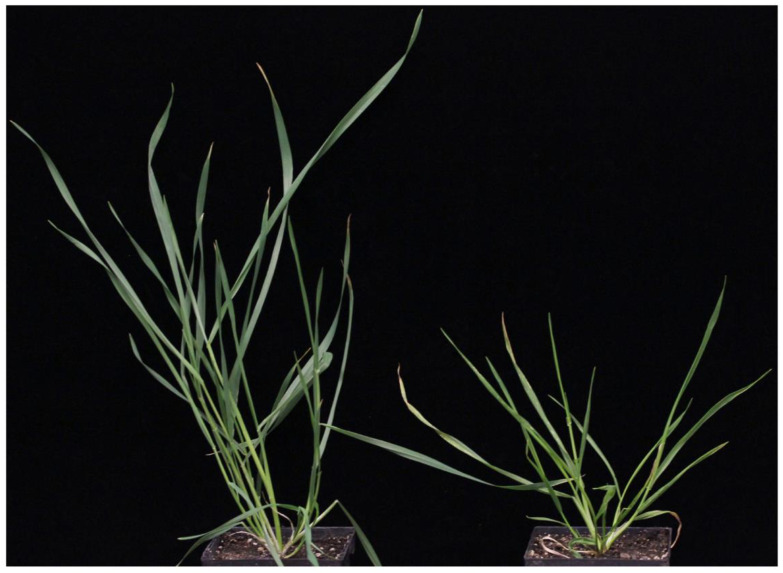
WDIV-infected Sonalika (**left**) and WL711 (**right**) wheat plants. Both plants are of the same age. The picture was taken at 21 days post infection.

Of the 12 plants (six WL711 and six Sonalika), six from WL711 and four from Sonalika tested positive for the virus. The virus-infected WL711 plants showed substantial retardation in height and biomass, whereas Sonalika plants showed mild to no symptoms (Figure 1). This is in agreement with the previous studies [10,18] confirming WL711 as a sensitive and Sonalika as a resistant cultivar for WDIV infection.

### 3.2. Transcriptome Changes

**Figure 2:**
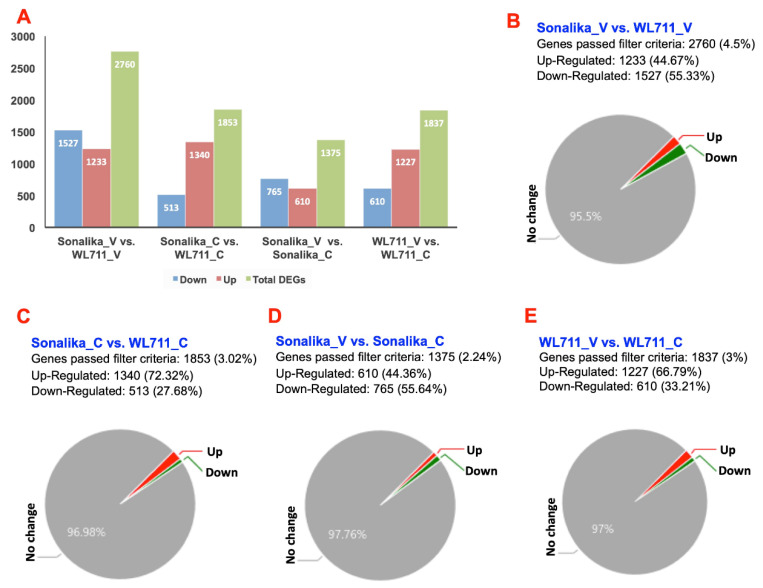
Differentially expressed transcripts. Panel (**A**) shows total number of differentially expressed transcripts (shown in green), up- (red) and down- (blue) regulated transcripts in different conditions (Sonalika_V vs. WL711_V, Sonalika_C vs. WL711_C, Sonalika_V vs. Sonalika_C and WL711_V vs. WL711_C). Panels (**B**–**E**) show percentage of genes that are differentially expressed in different conditions. Sonalika_V and WL711_V are virus-infected Sonalika and WL711 plants and the Sonalika_C and WL711_C are mock-inoculated control plants.

The transcriptome data generated from the virus-infected leaf tissues of Sonalika and WL711 (Sonalika_V and WL711_V) and control (Sonalika_C and WL711_C) plants were compared. A total of 2760, 1853, 1375, and 1837 genes were identified as differentially expressed (with a fold change of ≥2) in Sonalika_V vs. WL711_V, Sonalika_C vs. WL711_C, Sonalika_V vs. Sonalika_C and WL711_V vs. WL711_C, respectively (Figure 2A). The differentially expressed genes (DEGs) represented less than 5% of the total genes in all four conditions (Figure 2B–E), suggesting these genes’ possible involvement in stress response.

**Figure 3:**
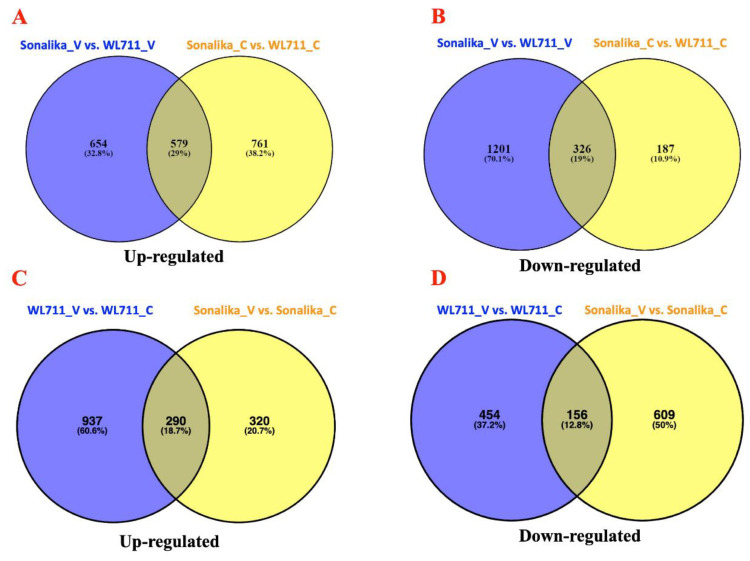
Venn diagram showing total number of up- and down-regulated transcripts (≥2-fold change; *p* ≤ 0.01) in virus-infected (Sonalika_V vs. WL711_V) and control (Sonalika_C vs. WL711_C) conditions (**A**,**B**) and WL711_V vs. WL711_C and Sonalika_V vs. Sonalika_C (**C**,**D**).

DEGs were further categorized into common and unique genes (Figure 3). A total of 579 and 326 genes were commonly up- and down-regulated in Sonalika_V vs. WL711_V and Sonalika_C vs. WL711_C, whereas a total of 654 and 1201 genes were up- and down-regulated uniquely in Sonalika infected with virus (Figure 3), which indicates possible involvement of these genes in stress response. In addition, Sonalika had 320 and 609 up- and down-regulated unique genes in Sonalika_V vs. Sonalika_C as compared to WL711_V vs. WL711_C (Figure 3), indicating differences at the genotype level for the two cultivars.

### 3.3. Functional Assessment of Differentially Expressed Genes

**Figure 4:**
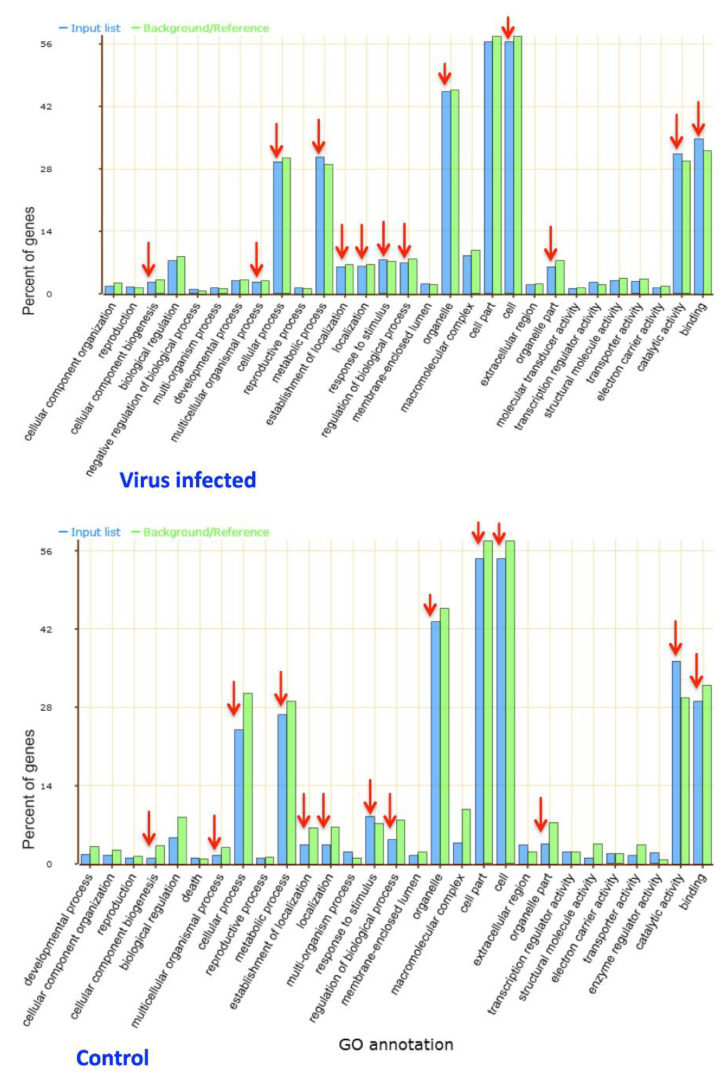
Bar graph representation of enrichment of gene ontology (GO) term for genes differentially expressed in mock-inoculated control and virus-infected Sonalika as compared to WL711. The X-axis represents GO annotation for predicted functions of the genes and the Y-axis shows percent of genes for each function. The green bars represent number of genes in reference list, whereas the blue bars represent the number of genes in the input list. Red arrows indicate the differences in percentage of genes in each functional category between the virus-infected and control samples.

The AgriGO categorization revealed differential expression of the genes related to binding, catalytic activity, cell and cellular process, metabolic process, response to stimulus, and regulation of biological process (Figure 4). The virus-infected Sonalika has a higher percentage of differentially expressed genes as compared to the mock-inoculated plants for the above-mentioned functions (Figure 4), which indicates the probable role of these genes in stress tolerance.

**Figure 5:**
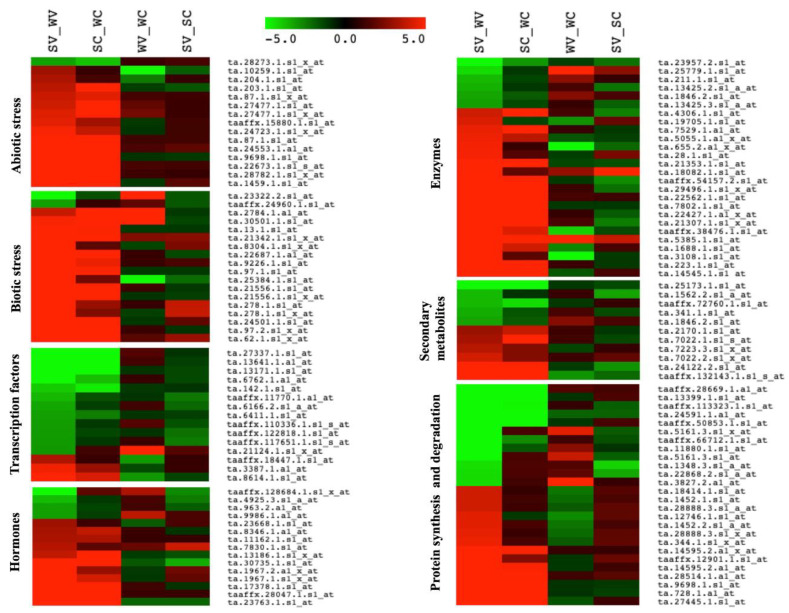
Heat maps of the significant transcripts involved in stress, transcription factors, hormone metabolism, enzymes, secondary metabolism, and protein synthesis and degradation. Transcripts with ≥5-fold change from SV vs. WV condition were used. Fold change of the same transcripts in other conditions (SC vs. WC, SV vs. SC and WV vs. WC) are compared. (SV) Sonalika infected with virus, (WV) WL711 infected with virus, (SC) mock-inoculated Sonalika and (WC) mock-inoculated WL711.

The expression heat map of DEGs revealed elevated transcription of several genes belonging to abiotic and biotic stress, enzymes, hormones, protein synthesis, and degradation in Sonalika_V vs. WL711_V (Figure 5) that might play a role in virus resistance. The transcription levels of the same genes were less significant in the control condition, Sonalika_C vs. WL711_C (Figure 5).

**Figure 6:**
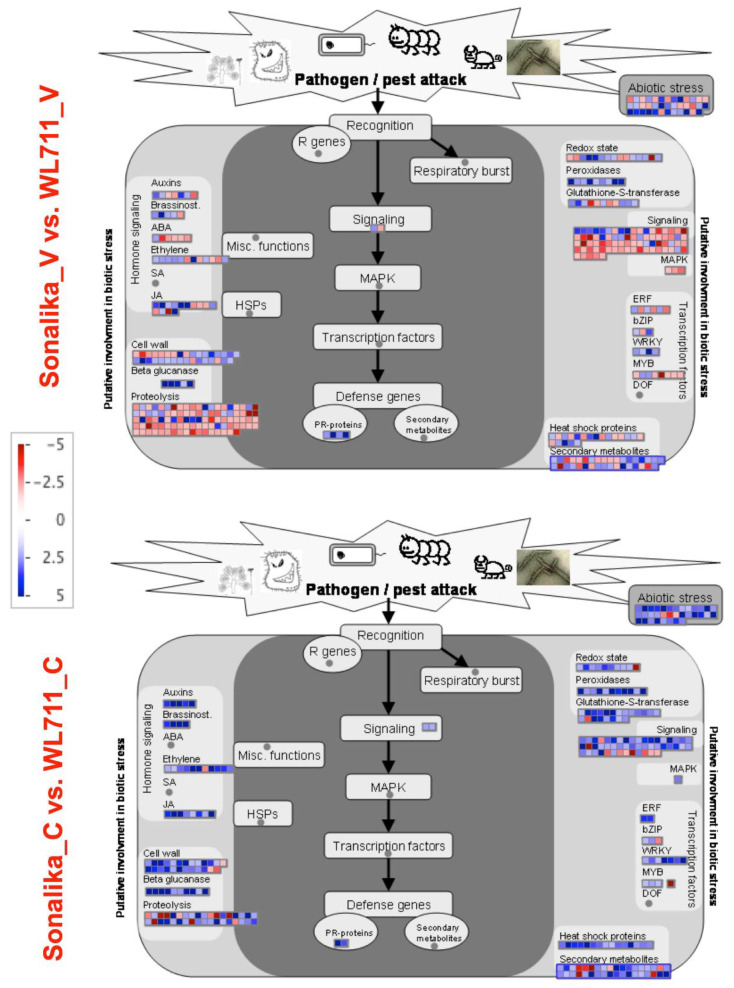
MapMan overview showing differentially expressed transcripts related to biotic and abiotic stress in Sonalika (Sonalika_V vs. WL711_V and Sonalika_C vs. WL711_C). Upregulated genes are shown by light to deep blue color boxes and the downregulated genes are shown by light to deep red color boxes. Color intensities show the level of expressions as indicated by the intensity bar on left. Genes involved in same functions are clubbed together. The transcripts with ≥2-fold change were used for the analysis.

Mapman categorization revealed genes related to abiotic stresses, signaling, secondary metabolism, proteolysis, hormone signaling, cell wall, transcription factors, enzymes, and PR proteins as differentially expressed in Sonalika_V vs. WL711_V and in Sonalika_C vs. WL711_C (Figure 6). Some of the genes that were upregulated in Sonalika_C vs. WL711_C were found downregulated in Sonalika_V vs. WL711_V and vice-versa (Figure 6), which indicates their expression in response to virus infection. A significant level of difference was observed in the accumulation of transcripts related to abiotic stresses, signaling, secondary metabolism, proteolysis, cell wall, hormone signaling, and transcription factors in Sonalika_V vs. Sonalika_C and in WL711_V vs. WL711_C (Figure S1). Most of the genes related to signaling and proteolysis were downregulated in Sonalika compared to WL711 (Figure S1). The differentially expressed genes related to abiotic stresses, signaling, PR proteins, and other functional categories described above are probably contributing towards observed virus resistance in Sonalika.

### 3.4. Differentially Expressed Pathways and Genes Involved in Resistance

**Figure 7:**
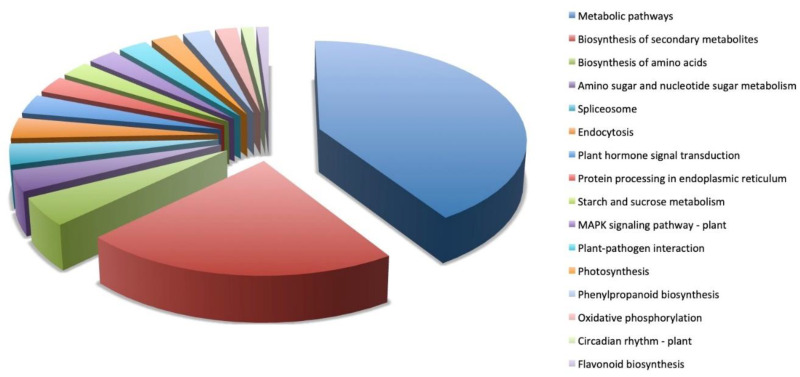
Classification of Kyoto Encyclopedia of Genes and Genomes (KEGG) pathways detected in the present analysis. The differentially expressed pathways in Sonalika in response to virus infection are shown here.

KEGG analysis using DEGs revealed diverse defense mechanisms in Sonalika (Figure 7). A large number of DEGs represented metabolic pathways and biosynthesis of secondary metabolites in the WDIV-infected Sonalika (Figure 7). Other DEGs were found to be involved in plant hormone signal transduction, plant–pathogen interaction, protein processing, MAPK signaling, photosynthesis, and starch and sucrose metabolism pathways (Figure 7).

### 3.5. MAPK Signaling Pathway

**Figure 8:**
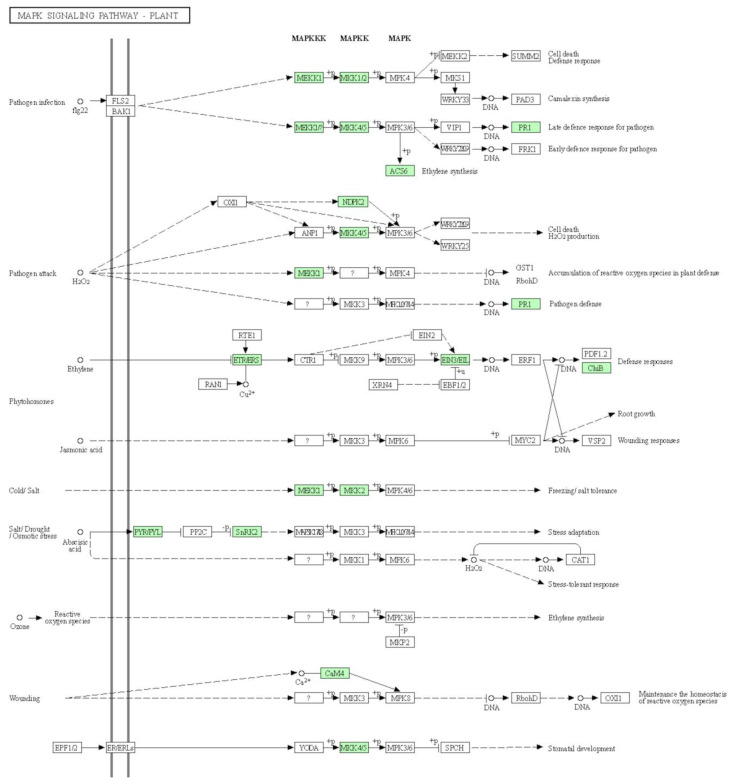
Kyoto Encyclopedia of Genes and Genomes (KEGG) pathway map analysis of DEGs involved in MAPK signaling pathway. Significant DEGs (log2 fold change ≥2 or ≤−2) are shown in green boxes, while the genes with non-significant expression (≥−2 or ≤2) are shown in uncolored boxes.

The mitogen-activated protein kinase (MAPK) pathway is evolutionarily conserved among eukaryotes and plays crucial roles in cellular processes, including adaption to biotic and abiotic stresses [24]. The present study documented 12 genes (green boxes in Figure 8) that were differentially expressed in the resistant line, Sonalika, in response to the virus infection. In one possible scenario, the interaction of mitogen-activated protein kinase kinase kinase 1 (MEKK1) with mitogen-activated protein kinase kinase 2 (MKK2) could have led to phosphorylation of MKK2 (Figure 8). Alternately, the interaction of MEKK1 with MKK4/5 led to phosphorylation of MKK4/5 and subsequent activation of pathogenesis-related protein 1 (PR1) protein, leading to a defense response and resistance in Sonalika. In another possible scenario, ethylene receptor (ETR/ERS) could have triggered phosphorylation of ethylene-insensitive protein 3 (EIN3) and expression of endochitinase B (ChiB), leading to a defense response and resistance (Figure 8). Other important genes that might have played a role in resistance in Sonalika are abscisic acid receptor (PYR/PYL), serine/threonine-protein kinase SRK2 (SnRK2), 1-aminocyclopropane-1-carboxylate synthase 6 (ACS6), and nucleoside-diphosphate kinase 2 (NDPK2), as predicted by the KEGG pathway analysis (Figure 8).

### 3.6. Plant–Pathogen Interaction Pathway

**Figure 9:**
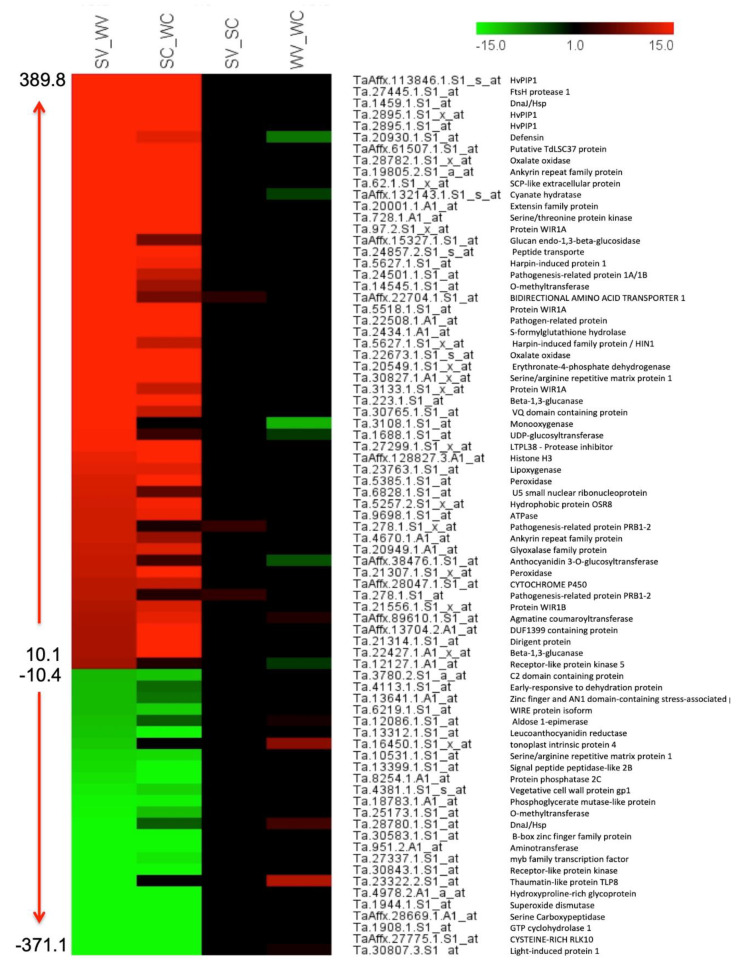
A heat-map of the transcripts ≥10 folds up- and down-regulated in SV vs. WV condition and their expression values in other conditions (SC vs. WC, SV vs. SC and WV vs. WC). Predicted functions of the transcripts are shown next to their probe IDs. (SV) Sonalika infected with virus, (WV) WL711 infected with virus, (SC) mock-inoculated Sonalika, and (WC) mock-inoculated WL711.

Plants have evolved multiple layers of defense systems against invading pathogens. The primary response is mediated via PAMP-triggered immunity (PTI). The binding of pathogen effectors triggers MAPK signaling pathway that activates defense genes such as PR1 (Figure S2). The differential expression of MEKK1 and MKK4/5 could have led to elevated expression of PR1 protein (Figure S2). Elevated expressions of several probe sets such as Ta.62.1.S1_x_at (38 fold), Ta.24501.1.S1_at (24 fold), Ta.278.1.S1_x_at (12 fold), and Ta.278.1.S1_at (11 fold) representing PR proteins in Sonalika in response to virus infection (Figure 9) could be contributing to virus resistance [25]. Other significantly expressed genes in the pathway were 3-ketoacyl-CoA synthase (KCS), disease resistance protein RAR1, heat shock protein 90 (HSP90), disease resistance protein RPM1, and pto-interacting protein 1 (Pti1) (Figure S2). R gene-mediated resistance is one of the best-characterized resistance responses in plants, where each R gene confers resistance to a specific pathogen [26,27]. The differential expression of multiple R genes such as PR1, RAR1, and RPM1 (Figure S2) shows the presence of intracellular surveillance resulting in disease resistance in Sonalika.

### 3.7. Plant Hormone Signal Transduction

Plant hormone signaling systems such as jasmonate (JA), salicylate (SA), abscisic acid (ABA), ethylene (ET), auxins, cytokinins, gibberellins, and brassinosteroids are critical in activation of the plant immune system [28]. KEGG pathway analysis revealed a total of 15 genes differentially expressed in the plant hormone signal transduction pathway, including the genes related to auxin, cytokinin, brassinosteroids, ABA, ET, and SA (shown in green boxes in Figure S3). The SA, JA, and ET levels increase upon pathogen infection and play a major role in response to biotic stress [29]. Three ET genes, ethylene receptor (ETR), ethylene-insensitive protein 3 (EIN3), and stress-induced MAPK (SIMK), and two SA related transcription factors, TGA and PR1, were found to be differentially expressed, which could be critical in determining viral infection resistance in Sonalika (Figure S3).

### 3.8. Putative Genes with the Highest Differential Expression and Their Role

We found several genes with significant up- (≥10 fold) or down-regulation (≤10 fold) in Sonalika in response to the virus infection (Figure 9). The gene with the highest upregulation was PLASMA MEMBRANE INTRINSIC PROTEIN 1 (PIP1) and downregulation was light-induced protein 1 (Figure 9). Three transcripts for PIP1 (TaAffx.113846.1.S1_s_at, Ta.2895.1.S1_x_at, and Ta.2895.1.S1_at) and one transcript for light-induced protein 1 (Ta.30807.3.S1_at) exhibited the highest upregulation and downregulation in Sonalika in comparison to WL711 (Figure 9). PIP1 is a member of the aquaporins (AQPs) family, which contributes to regulating water transport in response to drought and other abiotic stresses [30]. A transcript (Ta.30807.3.S1_at) representing light-induced protein 1 was found highly downregulated in Sonalika (Figure 9). Early light-induced protein (ELIP) has been reported to work as a photoprotectant [31]. We hypothesize that the elevated expression of PIP1 and downregulation of LIP1 in response to virus infection may be due to the cross-talk between the abiotic and biotic stresses in response to WDIV infection.

DnaJ proteins are molecular chaperones and respond to various environmental stresses. Overexpression of *SlDnaJ20* was reported to confer thermal tolerance in transgenic tomatoes, whereas suppression increased the heat sensitivity [32]. Elevated expression of a transcript (Ta.1459.1.S1_at) of DnaJ in virus infection points towards its probable role in biotic stress tolerance.

Plant defensins are small, cysteine-rich peptides that possess biological activity towards many organisms, including fungi [33]. We documented a highly upregulated transcript for defensin (Ta.20930.1.S1_at; Figure 9), which might contribute towards virus resistance in Sonalika.

Plants expressing oxalate oxidase (OXO) enzymes evoke defense responses by breaking down oxalate into CO_2_ and H_2_O_2_ [34]. We found two transcripts (Ta.28782.1.S1_x_at and Ta.22673.1.S1_s_at) that were significantly upregulated in Sonalika (Figure 9) and may be involved in virus resistance.

The ankyrin repeat protein family participates in multiple processes, including response to biotic and abiotic stresses by activating downstream defense signaling components [35]. We found two highly upregulated transcripts (Ta.19805.2.S1_a_at and Ta.4670.1.A1_at) representing the ankyrin repeat family, which is in agreement with the known role of ANK genes in achieving resistance to biotic and abiotic stresses.

Leucine-rich repeat extensin proteins have been reported to regulate salt tolerance in *Arabidopsis* [36]. One of the probeset (Ta.20001.1.A1_at), representing a transcript, showed higher upregulation (Figure 9) and may also contribute to virus resistance in Sonalika.

Serine-threonine kinase from *Capsicum annuum*, *CaDIK1*, has been reported to regulate drought tolerance by playing a role in attaching phosphate to the target protein. *CaDIK1*-silenced pepper plants were found to have reduced ABA sensitivity and drought hypersensitivity [37]. We found higher upregulation of a probe set (Ta.728.1.A1_at) representing serine-threonine kinase in Sonalika in response to virus infection, indicating a possible role in biotic stress as well.

Serine/arginine repetitive (SR) matrix proteins are major regulators of alternative splicing that generate proteomic diversity, which is crucial for plant stress responses [38,39,40]. We identified up- and down-regulated SR proteins (Ta.30827.1.A1_x_at, Ta.10531.1.S1_at, and Ta.6179.1.S1_at) in Sonalika, as compared to WL711 (Figure 9), which could be involved in virus resistance.

Beta-1,3-glucanases, also known as PR proteins, play a crucial role in plant defense against fungal pathogens. The expression of beta-1,3-glucanases in sugarcane (*ScGluD2*) was highly up-regulated in smut-resistant cultivars as compared to the susceptible cultivar [41]. The transcript levels of beta-1,3-glucanase genes (TaAffx.15327.1.S1_at, Ta.223.1.S1_at, and Ta.22427.1.A1_x_at) were highly upregulated in Sonalika as compared to WL711 (Figure 9), which indicates its role in virus resistance as well.

O-methyltransferases (OMTs) are an essential group of enzymes that catalyze the transfer of a methyl group from S-adenosyl-L-methionine to their acceptor substrates and have been reported to play roles in abiotic and biotic stress tolerance [42,43,44]. Interestingly, one putative transcript for O-methyltransferase was highly upregulated (Ta.14545.1.S1_at) and one was downregulated (Ta.25173.1.S1_at) in Sonalika (Figure 9), suggesting a role in virus resistance.

Conversion of H_2_O_2_ into H_2_O is catalyzed by ascorbate peroxidase, which prevents plant tissue from damage due to ROS. Transgenic plants overexpressing ascorbate peroxidase have been shown to protect plants from oxidative damage [45,46]. Multiple transcripts of peroxidase genes (Ta.5385.1.S1_at, Ta.21307.1.S1_x_at) were highly upregulated in Sonalika (Figure 9), suggesting their role in stress tolerance.

Glycosyl hydrolases play roles in response to biotic and abiotic stresses and cell wall remodeling [47]. Multiple transcripts for glycosyl hydrolase (TaAffx.15327.1.S1_at, Ta.223.1.S1_at, and Ta.22427.1.A1_x_at) were accumulated at a very high level in Sonalika, in comparison to WL711 (Figure 9), indicating its significance in virus infection as well.

The high-level expression of cytochrome P450 transcripts (Ta.3108.1.S1_at and TaAffx.28047.1.S1_at) in Sonalika (Figure 9) is in agreement with the previous studies related to biotic and abiotic stresses [15,48].

The domain of unknown function 26 (DUF26), a receptor-like kinase (RLK), plays an important role in the regulation of pathogen defense and programmed cell death [49]. A transcript (TaAffx.27775.1.S1_at) representing DUF26 was highly downregulated (Figure 9), which coincides with a previous report [49].

### 3.9. Uncharacterized Protein

Several transcripts of unknown function were detected, exhibiting differential expression of 10-fold or more (Table S2). Differential expression of these transcripts in response to the virus infection indicates their possible role in virus resistance. A further investigation on the functional characterization of these genes may confer their role in stress resistance.

### 3.10. Quantitative RT-PCR

The relative fold change in expression of five randomly chosen genes and three uncharacterized proteins (Table S1) was examined. The expression profile of these genes was in agreement with the microarray results (Figure S4). Hence, q-RT PCR results validated the microarray data. However, the differential transcriptional investigation should be followed up with reverse genetics studies.

## 4. Conclusions

The microarray-based comparative transcriptome profiling of two contrasting genotypes for virus resistance identified a significant number of differentially expressed transcripts related to the stress response in Sonalika. The investigation identified critical pathways and genes that could confer virus resistance in Sonalika. Activation of the MAPK pathway might contribute to virus resistance via the action of PR1 protein and ChiB. Moreover, the differential expression of multiple R genes such as PR1, RAR1, and RPM1 represents robust intracellular surveillance that could confer virus resistance in Sonalika. The upregulation of PIP1 and downregulation of LIP1 suggest cross-talk between the abiotic and biotic stresses and are subsequently crucial for resistance in Sonalika. Other differentially expressed genes including DnaJ, defensins, oxalate oxidase, ankyrin repeat protein, serine-threonine kinase, SR proteins, beta-1,3-glucanases, O-methyltransferases, glycosyl hydrolase, and DUF26, may also be critical in defense responses. Interestingly, significant expression of many transcripts of unknown functions was detected in Sonalika. Their possible role in stress tolerance should be investigated by functionally characterizing them. A future study focused on silencing these differentially expressed genes, using virus-induced gene silencing or RNAi, should be undertaken and their role in WDIV resistance verified. The outcome of this study could be utilized as the foundation for programs aimed at functional genomics, marker development, and incorporating virus resistance in wheat.

## Data Availability

The microarray raw data have been deposited at NCBI in the Gene Expression Omnibus (GEO) database under the accession number GSE179675.

## Supplementary Materials

The following are available online at https://www.mdpi.com/article/10.3390/life11090955/s1, Figure S1: MapMan overview showing differentially expressed probesets related to biotic and abiotic stress in Sonalika_V vs. Sonalika_C and WL711_V vs. WL711_C. Up-regulated genes are shown by light to deep blue color boxes, and the down-regulated genes are shown by light to deep red color boxes. Color intensity show the level of expressions as indicated by the intensity bar. Genes involved in same functions are clubbed together, Figure S2: Kyoto Encyclopedia of Genes and Genomes (KEGG) pathway map analysis of DEGs involved in plant-pathogen interaction (PPI). PPI is one of the significant pathways detected after virus infection and determines resistance or susceptibility. Significant DEGs (log2 fold change ≥2 or ≤−2) are shown in green boxes, while the genes with non-significant expression (≥−2 or ≤2) are shown in black boxes, Figure S3: Kyoto Encyclopedia of Genes and Genomes (KEGG) pathway map analysis of DEGs involved in hormone signal transduction, which is one of the significant pathways detected after virus infection, and determines resistance or susceptibility. Significant DEGs (log2 fold change ≥2 or ≤−2) are shown in green boxes, while the genes with non-significant expression (≥−2 or ≤2) are shown in black boxes, Figure S4: Quantitative real-time PCR based validation of gene expression. The expression values estimated by microarray (micro) are compared with real-time (RT). The conditions are SV_WV (Sonalika virus vs. WL711 virus) and SC_WC (Sonalika control vs. WL711 control). Table S1: List of primers used in real-time PCR assays, Table S2: Uncharacterized protein in our dataset with more than 10 fold differential expression in response to virus infection.

## References

[1] Zerbini F.M., Briddon R.W., Idris A., Martin D.P., Moriones E., Navas-Castillo J., Rivera-Bustamante R., Roumagnac P., Varsani A., ICTV Report Consortium (2017). ICTV Virus Taxonomy Profile: Geminiviridae. J. Gen. Virol..

[2] Miozzi L., Napoli C., Sardo L., Accotto G.P. (2014). Transcriptomics of the interaction between the monopartite phloem-limited geminivirus tomato yellow leaf curl Sardinia virus and *Solanum lycopersicum* highlights a role for plant hormones, autophagy and plant immune system fine tuning during infection. PLoS ONE.

[3] Góngora-Castillo E., Ibarra-Laclette E., Trejo-Saavedra D.L., Rivera-Bustamante R.F. (2012). Transcriptome analysis of symptomatic and recovered leaves of geminivirus-infected pepper (*Capsicum annuum*). Virol. J..

[4] Pierce E.J., Rey M.E. (2013). Assessing global transcriptome changes in response to South African cassava mosaic virus [ZA-99] infection in susceptible Arabidopsis thaliana. PLoS ONE.

[5] Ascencio-Ibáñez J.T., Sozzani R., Lee T.J., Chu T.M., Wolfinger R.D., Cella R., Hanley-Bowdoin L. (2008). Global analysis of Arabidopsis gene expression uncovers a complex array of changes impacting pathogen response and cell cycle during geminivirus infection. Plant Physiol..

[6] Wu M., Ding X., Fu X., Lozano-Duran R. (2019). Transcriptional reprogramming caused by the geminivirus Tomato yellow leaf curl virus in local or systemic infections in *Nicotiana benthamiana*. BMC Genom..

[7] Naqvi R.Z., Zaidi S.S., Akhtar K.P., Strickler S., Woldemariam M., Mishra B., Mukhtar M.S., Scheffler B.E., Scheffler J.A., Jander G. (2017). Transcriptomics reveals multiple resistance mechanisms against cotton leaf curl disease in a naturally immune cotton species, *Gossypium arboreum*. Sci. Rep..

[8] Zaidi S.S., Naqvi R.Z., Asif M., Strickler S., Shakir S., Shafiq M., Khan A.M., Amin I., Mishra B., Mukhtar M.S. (2020). Molecular insight into cotton leaf curl geminivirus disease resistance in cultivated cotton (*Gossypium hirsutum*). Plant Biotechnol. J..

[9] Kumar J., Singh S.P., Kumar J., Tuli R. (2012). A novel mastrevirus infecting wheat in India. Arch. Virol..

[10] Kumar J., Kumar J., Singh S.P., Tuli R. (2014). Association of satellites with a mastrevirus in natural infection: Complexity of Wheat dwarf India virus disease. J. Virol..

[11] Kumar J., Kumar S., Kianian S.F. (2020). The Wheat dwarf India virus-betasatellite complex has a wider host range than previously reported. Plant Health Prog..

[12] Fondong V.N. (2013). Geminivirus protein structure and function. Mol. Plant Pathol..

[13] Marathe R., Guan Z., Anandalakshmi R., Zhao H., Dinesh-Kumar S.P. (2004). Study of *Arabidopsis thaliana* resistome in response to cucumber mosaic virus infection using whole genome microarray. Plant Mol. Biol..

[14] Kaur H., Yadav C., Alatar A., Faisal M., Jyothsna P., Malathi V.G., Praveen S. (2014). Gene expression changes in tomato during symptom development in response to leaf curl virus infection. J. Plant Biochem. Biotechnol..

[15] Narusaka Y., Narusaka M., Seki M., Umezawa T., Ishida J., Nakajima M., Enju A., Shinozaki K. (2004). Crosstalk in the responses to abiotic and biotic stresses in Arabidopsis: Analysis of gene expression in cytochrome P450 gene superfamily by cDNA microarray. Plant Mol. Biol..

[16] Atkinson N.J., Urwin P.E. (2012). The interaction of plant biotic and abiotic stresses: From genes to the field. J. Exp. Bot..

[17] Liu L., Chung H.Y., Lacatus G., Baliji S., Ruan J., Sunter G. (2014). Altered expression of Arabidopsis genes in response to a multifunctional geminivirus pathogenicity protein. BMC Plant Biol..

[18] Kumar J., Kumar J., Singh S., Shukla V., Singh S.P., Tulị R. (2015). Prevalence of Wheat dwarf India virus in wheat in India. Curr. Sci..

[19] Jun A.S., Liu S.H., Koo E.H., Do D.V., Stark W.J., Gottsch J.D. (2001). Microarray analysis of gene expression in human donor corneas. Arch. Ophthalmol..

[20] Letwin N.E., Kafkafi N., Benjamini Y., Mayo C., Frank B.C., Luu T., Lee N.H., Elmer G.I. (2006). Combined application of behavior genetics and microarray analysis to identify regional expression themes and gene-behavior associations. J. Neurosci..

[21] Roy Choudhury D., Small C., Wang Y., Mueller P.R., Rebel V.I., Griswold M.D., McCarrey J.R. (2010). Microarray-based analysis of cell-cycle gene expression during spermatogenesis in the mouse. Biol. Reprod..

[22] Saeed A.I., Bhagabati N.K., Braisted J.C., Liang W., Sharov V., Howe E.A., Li J., Thiagarajan M., White J.A., Quackenbush J. (2006). TM4 microarray software suite. Methods Enzymol..

[23] Kanehisa M., Goto S. (2000). KEGG: Kyoto Encyclopedia of Genes and Genomes. Nucleic Acids Res..

[24] Sinha A.K., Jaggi M., Raghuram B., Tuteja N. (2011). Mitogen-activated protein kinase signaling in plants under abiotic stress. Plant Signal Behav..

[25] Fang L.J., Qin R.L., Liu Z., Liu C.R., Gai Y.P., Ji X.L. (2019). Expression and functional analysis of a PR-1 Gene, MuPR1, involved in disease resistance response in mulberry (*Morus multicaulis*). J. Plant Interact..

[26] Elvira M.I., Galdeano M.M., Gilardi P., García-Luque I., Serra M.T. (2008). Proteomic analysis of pathogenesis-related proteins (PRs) induced by compatible and incompatible interactions of pepper mild mottle virus (PMMoV) in Capsicum chinense L3 plants. J. Exp. Bot..

[27] Wu L., Chen H., Curtis C., Fu Z.Q. (2014). Go in for the kill: How plants deploy effector-triggered immunity to combat pathogens. Virulence.

[28] Verma V., Ravindran P., Kumar P.P. (2016). Plant hormone-mediated regulation of stress responses. BMC Plant Biol..

[29] Bari R., Jones J.D. (2009). Role of plant hormones in plant defence responses. Plant Mol. Biol..

[30] Vandeleur R.K., Mayo G., Shelden M.C., Gilliham M., Kaiser B.N., Tyerman S.D. (2009). The role of plasma membrane intrinsic protein aquaporins in water transport through roots: Diurnal and drought stress responses reveal different strategies between isohydric and anisohydric cultivars of grapevine. Plant Physiol..

[31] Lee J.W., Lee S.H., Han J.W., Kim G.H. (2020). Early light-inducible protein (ELIP) can enhance resistance to cold-induced photooxidative stress in *Chlamydomonas reinhardtii*. Front. Physiol..

[32] Wang G., Cai G., Xu N., Zhang L., Sun X., Guan J., Meng Q. (2019). Novel DnaJ protein facilitates thermotolerance of transgenic tomatoes. Int. J. Mol. Sci..

[33] Vriens K., Cammue B.P., Thevissen K. (2014). Antifungal plant defensins: Mechanisms of action and production. Molecules.

[34] Fang W., Jie H., Yan Y., Hao Z., Wu R., Tian B., Cao G., Xin Z. (2015). Transgenic *Arabidopsis thaliana* expressing a wheat oxalate oxidase exhibits hydrogen peroxide related defense response. J. Integr. Agric..

[35] Lopez-Ortiz C., Peña-Garcia Y., Natarajan P., Bhandari M., Abburi V., Dutta S.K., Yadav L., Stommel J., Nimmakayala P., Reddy U.K. (2020). The ankyrin repeat gene family in *Capsicum* spp.: Genome-wide survey, characterization and gene expression profile. Sci. Rep..

[36] Zhao C., Zayed O., Yu Z., Jiang W., Zhu P., Hsu C.C., Zhang L., Tao W.A., Lozano-Durán R., Zhu J.K. (2018). Leucine-rich repeat extensin proteins regulate plant salt tolerance in *Arabidopsis*. Proc. Natl. Acad. Sci. USA.

[37] Lim J., Lim C.W., Lee S.C. (2020). Pepper novel serine-threonine kinase CaDIK1 regulates drought tolerance via modulating ABA sensitivity. Front. Plant Sci..

[38] Tanabe N., Yoshimura K., Kimura A., Yabuta Y., Shigeoka S. (2007). Differential expression of alternatively spliced mRNAs of Arabidopsis SR protein homologs, atSR30 and atSR45a, in response to environmental stress. Plant Cell Physiol..

[39] Duque P. (2011). A role for SR proteins in plant stress responses. Plant Signal Behav..

[40] Yoshimura K., Mori T., Yokoyama K., Koike Y., Tanabe N., Sato N., Takahashi H., Maruta T., Shigeoka S. (2011). Identification of alternative splicing events regulated by an Arabidopsis serine/arginine-like protein, atSR45a, in response to high-light stress using a tiling array. Plant Cell Physiol..

[41] Su Y., Wang Z., Liu F., Li Z., Peng Q., Guo J., Xu L., Que Y. (2016). Isolation and Characterization of ScGluD2, a New Sugarcane beta-1,3-Glucanase D Family Gene Induced by *Sporisorium scitamineum*, ABA, H_2_O_2_, NaCl, and CdCl_2_ Stresses. Front. Plant Sci..

[42] Hafeez A., Gě Q., Zhāng Q., Lǐ J., Gōng J., Liú R., Shí Y., Shāng H., Liú À., Iqbal M.S. (2021). Multi-responses of O-methyltransferase genes to salt stress and fiber development of Gossypium species. BMC Plant Biol..

[43] Uchida K., Sawada Y., Ochiai K., Sato M., Inaba J., Hirai M.Y. (2020). Identification of a unique type of isoflavone O-Methyltransferase, GmIOMT1, based on multi-omics analysis of soybean under biotic stress. Plant Cell Physiol..

[44] Wang M., Zhu X., Wang K., Lu C., Luo M., Shan T., Zhang Z. (2018). A wheat caffeic acid 3-O-methyltransferase TaCOMT-3D positively contributes to both resistance to sharp eyespot disease and stem mechanical strength. Sci. Rep..

[45] Shigeoka S., Ishikawa T., Tamoi M., Miyagawa Y., Takeda T., Yabuta Y., Yoshimura K. (2002). Regulation and function of ascorbate peroxidase isoenzymes. J. Exp. Bot..

[46] Caverzan A., Passaia G., Rosa S.B., Ribeiro C.W., Lazzarotto F., Margis-Pinheiro M. (2012). Plant responses to stresses: Role of ascorbate peroxidase in the antioxidant protection. Genet. Mol. Biol..

[47] Opassiri R., Pomthong B., Onkoksoong T., Akiyama T., Esen A., Ketudat Cairns J.R. (2006). Analysis of rice glycosyl hydrolase family 1 and expression of Os4bglu12 beta-glucosidase. BMC Plant Biol..

[48] Devi B.S.R., Kim Y.J., Sathiyamoorthy S., Khorolragchaa A., Gayathri S., Parvin S., Yang D.U., Selvi S.K., Lee O.R., Lee S. (2011). Classification and characterization of putative cytochrome P450 genes from Panax ginseng C.A. Meyer. Biochemistry.

[49] Wrzaczek M., Brosché M., Salojärvi J., Kangasjärvi S., Idänheimo N., Mersmann S., Robatzek S., Karpiński S., Karpińska B., Kangasjärvi J. (2010). Transcriptional regulation of the CRK/DUF26 group of receptor-like protein kinases by ozone and plant hormones in Arabidopsis. BMC Plant Biol..

