# Supplementary material for: Study of Triticum aestivum Resistome in Response to Wheat dwarf India Virus Infection
Source: Life (Basel). 2021 Sep 13;11(9):955. doi: 10.3390/life11090955 (PMC8469153; doi:10.3390/life11090955)
Supplement: Supplementary file 1 [file life-11-00955-s001.zip › life-1359766-supplementary.pdf]

life

MDPI

Article

Study of *Triticum aestivum* Resistome in Response to *Wheat Dwarf India Virus* Infection

Jitendra Kumar [1,2], Krishan Mohan Rai [3], Shahryar F. Kianian [4,*] and Sudhir P. Singh [1,5,*]

[1] National Agri-Food Biotechnology Institute, SAS Nagar, Mohali 140306, India
[2] Department of Agronomy and Plant Genetics, University of Minnesota, St. Paul, MN 55108, USA; jkumar@umn.edu
[3] Department of Microbial and Plant Biology, University of Minnesota, St. Paul, MN 55108, USA; Kmohanra@umn.edu
[4] USDA-ARS Cereal Disease Laboratory, St. Paul, MN 55108, USA
[5] Center of Innovative and Applied Bioprocessing, SAS Nagar, Mohali 140306, India
* Correspondence: shahryar.kianian@usda.gov (S.F.K.); pratapsudhir@gmail.com (S.P.S.)

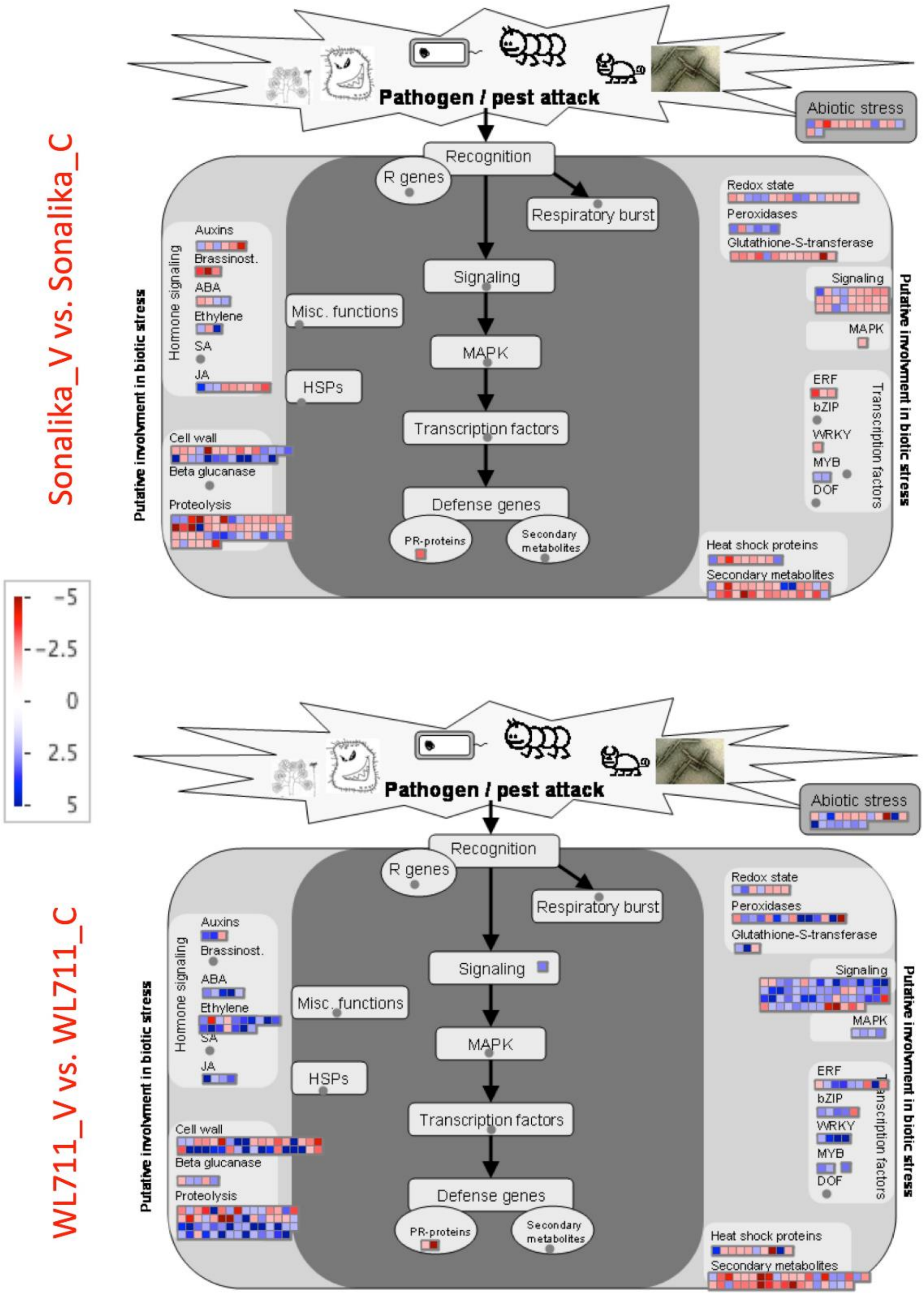

Figure S1. MapMan overview showing differentially expressed probesets related to biotic and abiotic stress in Sonalika_V vs. Sonalika_C and WL711_V vs. WL711_C. Up-regulated genes are shown by light to deep blue color boxes and the down-regulated genes are shown by light to deep red color boxes. Color intensity show the level of expressions as indicated by the intensity bar. Genes involved in same functions are clubbed together.

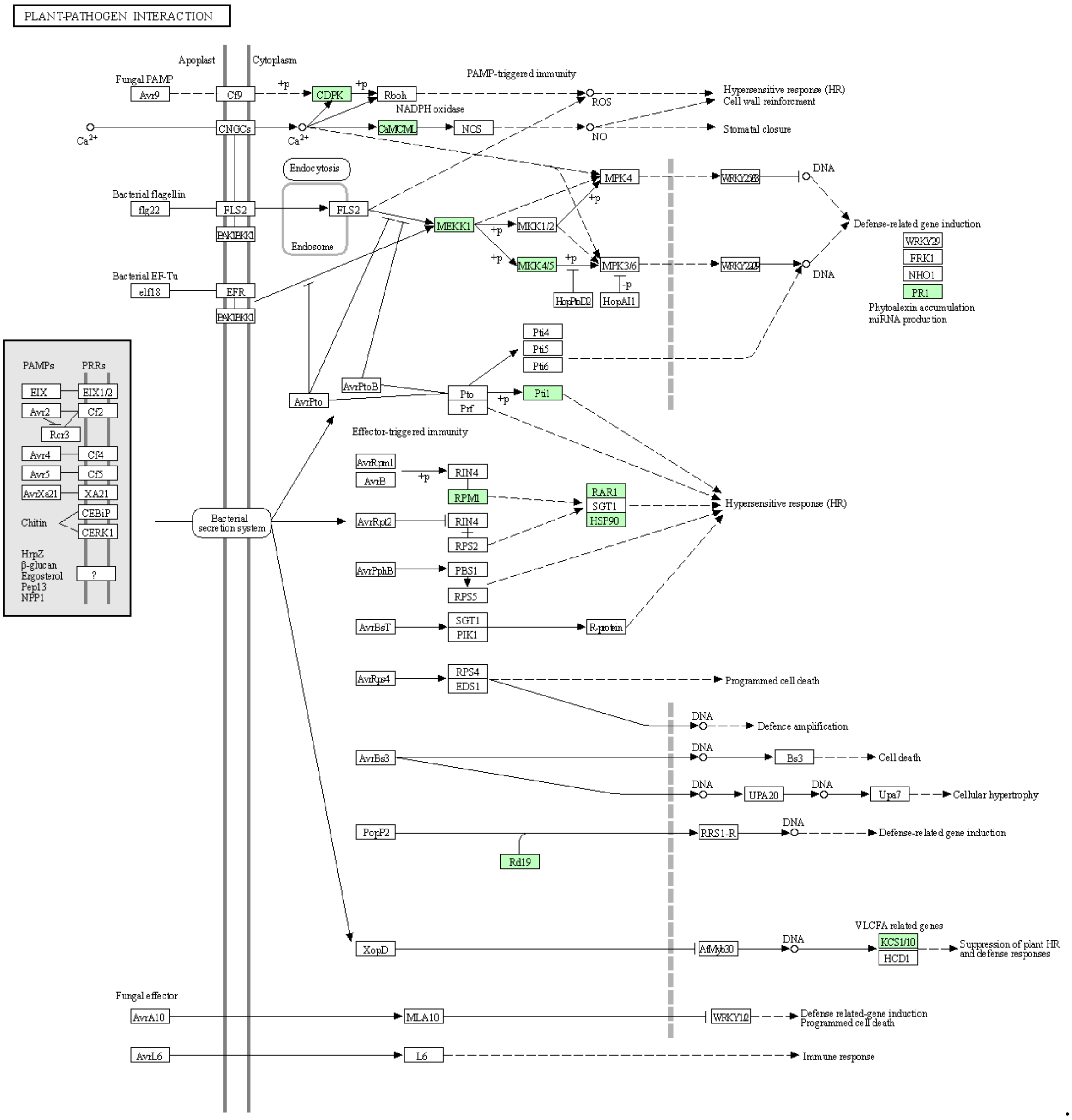

Figure S2. Kyoto Encyclopedia of Genes and Genomes (KEGG) pathway map analysis of DEGs involved in plant-pathogen interaction (PPI). PPI is one of the significant pathways detected after virus infection and determines resistance or susceptibility. Significant DEGs (log2 fold change ≥ 2 or ≤ - 2) are shown in green boxes, while the genes with non-significant expression (≥ - 2 or ≤ 2) are shown in black boxes.

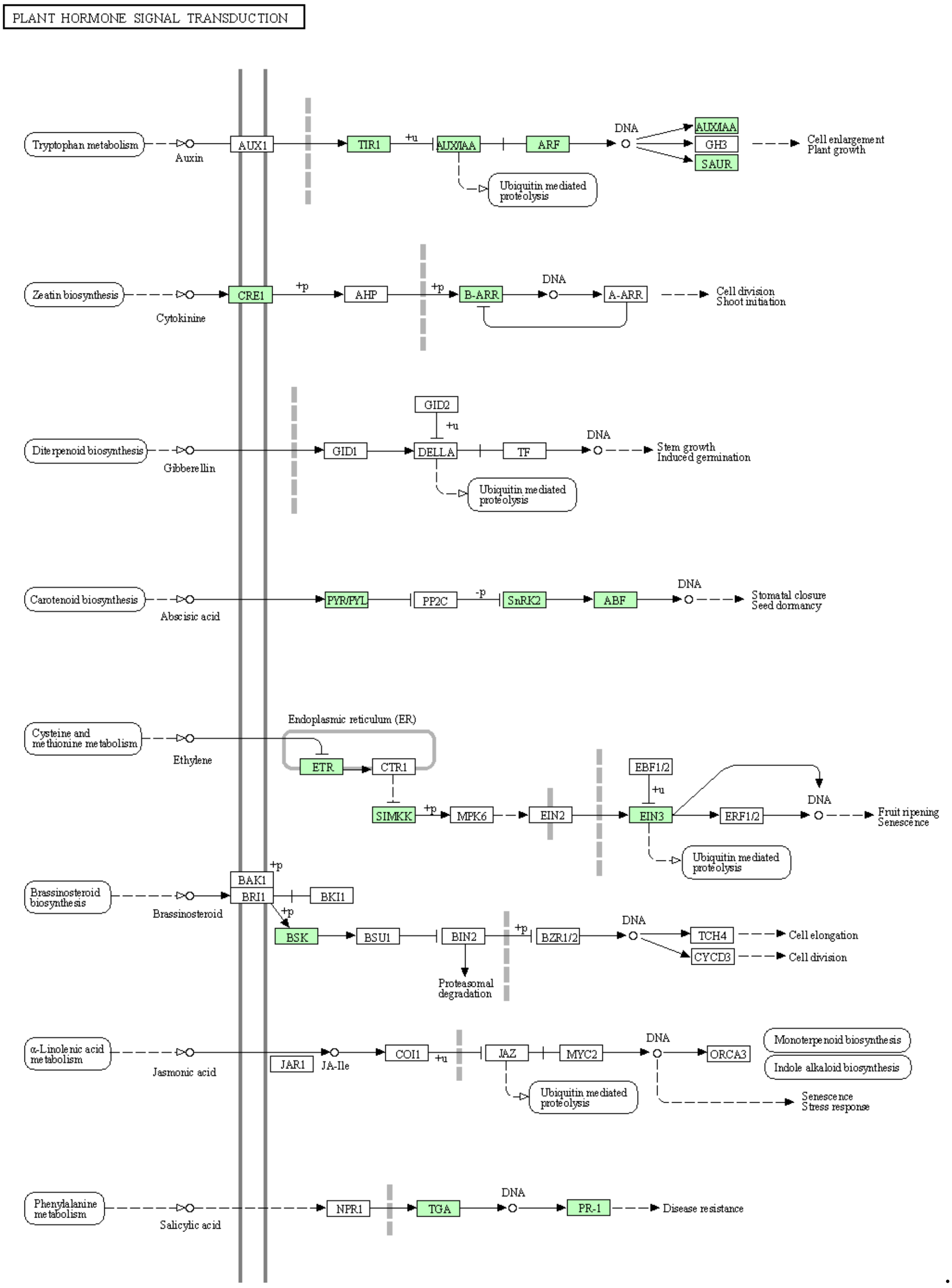

Figure S3. Kyoto Encyclopedia of Genes and Genomes (KEGG) pathway map analysis of DEGs involved in hormone signal transduction, which is one of the significant pathways detected after virus infection, and determines resistance or susceptibility. Significant DEGs (log2 fold change ≥ 2 or ≤ - 2) are shown in green boxes, while the genes with non-significant expression (≥ - 2 or ≤ 2) are shown in black boxes.

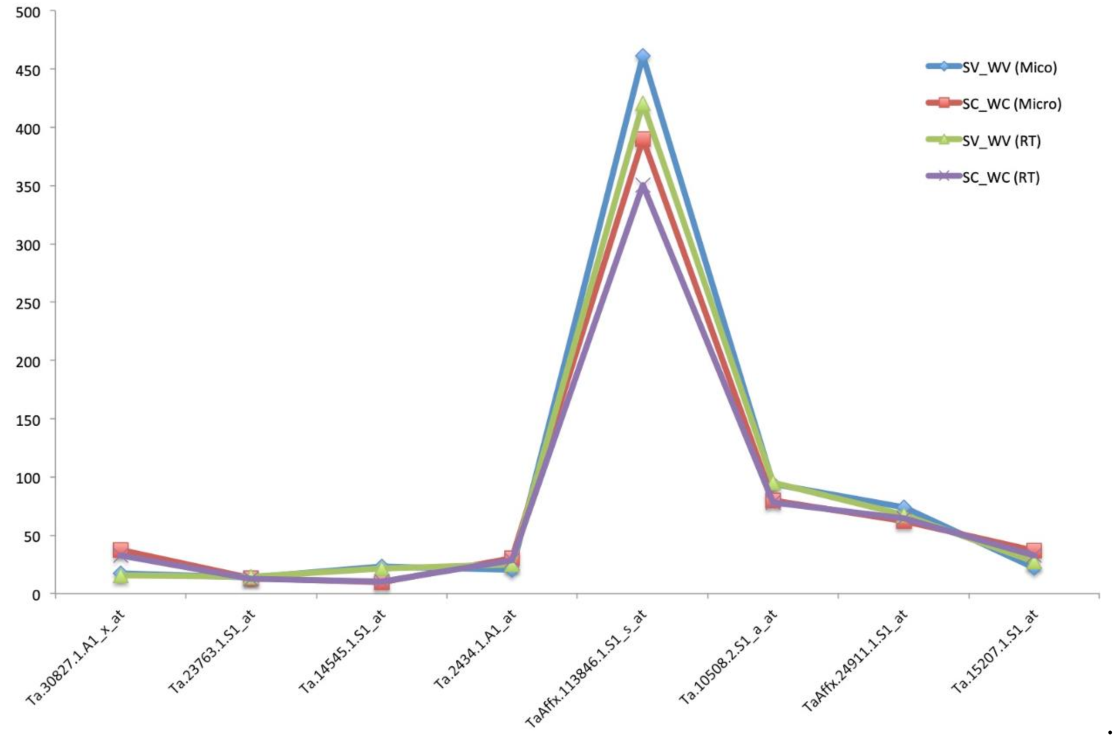

Figure S4. Quantitative real-time PCR based validation of gene expression. The expression values estimated by microarray (micro) are compared with real-time (RT). The conditions are SV_WV (Sonalika virus vs. WL711 virus) and SC_WC (Sonalika control vs. WL711 control).

Table S1. List of primers used in real-time PCR assays.

Probe ID/Gene Name	Forward Primer	Reverse Primer
	Randomly selected genes	
Ta.30827.1.A1_x_at	CCGTGACAGGGCTAGGTAAA	ATGGCCCAGTCATTATCTGC
Ta.23763.1.S1_at	CTCCTCCGCCAGCAGTTC	CGGCATGATGCTCCTCAG
Ta.14545.1.S1_at	CACATCGAACGAGACCAAGA	GAGGCCAGCTCGGACAAG
Ta.2434.1.A1_at	GCACGGTGCACTGACTATCT	TGCATCGTATTCCTCCCATT
TaAffx.113846.1.S1_s_at	CGCGACTAGTCAGCCAATC	GAGAGGGACAGAGGACGGTA
	Uncharacterized proteins	
Ta.10508.2.S1_a_at	GCGCATGAGCAAGAGTTACC	GTTCAGGGCTGGACACAAAT
TaAffx.24911.1.S1_at	TCGAATGCTGCTGAAACAAG	GCAACACATCATACGGCATC
Ta.15207.1.S1_at	CCATGGCTATGGAGAGGTTC	CATCTGCTTGCCAGTGAAGA
	Internal control	
EF-1α	GCTGTCAAGTTTGCTGAGATCC	GTACTGAGCGAAGGTCTCCAC

Table S2. Uncharacterized protein in our dataset with more than 10 fold differential expression in response to virus infection.

UniProt Desc	ID	SV_WV
**Uncharacterized protein**	TaAffx.103568.1.S1_at	98.11
	Ta.10508.2.S1_a_at	94.31
	TaAffx.24911.1.S1_at	74.19
	Ta.20262.2.S1_at	64.71
	Ta.22662.1.S1_at	56.77
	Ta.23165.2.S1_x_at	51.56
	TaAffx.100436.1.S1_at	51.27
	Ta.14446.1.A1_at	42.14
	Ta.883.1.S1_at	41.98
	Ta.23165.3.S1_x_at	33.3
	TaAffx.12907.1.A1_at	29.87
	TaAffx.74284.1.S1_at	26.14
	Ta.787.1.S1_x_at	25.74
	Ta.22462.1.S1_at	22.32
	Ta.15207.1.S1_at	22.05
	Ta.28842.1.S1_at	21.56
	Ta.896.3.S1_a_at	19
	Ta.21376.2.A1_a_at	18.31
	Ta.1112.1.S1_at	17.13
	Ta.21376.1.S1_at	16.81
	Ta.23904.1.S1_at	16.06
	Ta.17295.1.S1_at	15.4
	Ta.30770.1.S1_at	15.1
	Ta.7124.1.A1_at	14.57
	Ta.22230.1.S1_at	14.5
	TaAffx.28007.1.S1_at	14.37
	Ta.21996.1.S1_at	13.99
	Ta.9590.1.S1_at	13.76
	Ta.28983.2.S1_x_at	13.45
	TaAffx.27177.1.S1_at	13.25
	Ta.28983.2.S1_at	12.99
	Ta.22966.3.S1_at	12.57
	TaAffx.93984.1.S1_at	11.83
	Ta.11967.1.S1_at	11.13
	Ta.4606.1.A1_at	11.09
	Ta.12102.1.S1_a_at	10.89
	TaAffx.119440.1.A1_at	-10.41
	Ta.16930.1.S1_at	-10.81
	TaAffx.4964.1.S1_at	-10.83
	TaAffx.97191.1.S1_at	-10.87
	TaAffx.92234.1.A1_at	-11.28
	Ta.26429.1.S1_at	-13
	TaAffx.56231.1.S1_at	-13.05
	Ta.25828.1.A1_at	-13.92
	Ta.6254.1.S1_at	-14.21
	Ta.25891.1.S1_at	-14.59
	TaAffx.25418.1.S1_at	-15.15
	Ta.3242.1.A1_a_at	-15.6

	Ta.16175.1.A1_x_at	-15.78
	Ta.6179.1.S1_at	-15.81
	Ta.15173.1.A1_at	-16.26
	Ta.12024.1.A1_at	-19.46
	TaAffx.50281.1.S1_at	-20.01
	Ta.6515.1.S1_at	-20.52
	Ta.3527.1.S1_at	-20.59
	Ta.30500.1.S1_at	-20.6
	TaAffx.82405.1.S1_s_at	-20.6
	Ta.23352.1.S1_at	-22.1
	Ta.6645.1.A1_at	-22.51
	Ta.3830.2.S1_x_at	-23.02
	Ta.14725.1.S1_at	-27.09
	TaAffx.107501.1.S1_at	-34.07
	Ta.9531.1.A1_at	-36.35
	TaAffx.58902.1.S1_x_at	-41.39
	Ta.3242.3.A1_at	-51.69
	Ta.25193.1.S1_at	-52.47
	Ta.3830.1.S1_a_at	-53.95
	Ta.8331.1.S1_at	-55.26
	TaAffx.56616.1.S1_at	-76.83
	Ta.3242.1.A1_at	-107.34
